# Undiagnosed Pulmonary Tuberculosis Among Incarcerated Individuals and Its Overlooked Transmission Risk for the Community in Central Ethiopia

**DOI:** 10.1155/cjid/4170420

**Published:** 2025-08-11

**Authors:** Tedegn Teketel, Feleke Doyore Agide, Yohannes Yirga, Tadesse Hamdalla, Gizachew Beykaso

**Affiliations:** ^1^Department of Diseases Prevention and Control, Lemo Woreda Health Office, Hadiya Zone, Hossana, Central Ethiopia Region, Ethiopia; ^2^School of Public Health, College of Medicine and Health Science, Wachemo University, P.O. Box 667, Hossana, Ethiopia; ^3^Department of Public Health, Hossana College of Health Science, P.O. Box 159, Hossana, Central Ethiopia Region, Ethiopia

**Keywords:** Central Ethiopia, GeneXpert, incarcerated individuals, PTB, risk factors

## Abstract

**Background:** Tuberculosis (TB) remains a major public health problem globally, particularly in resource-limited settings where deprived ventilation, overcrowding, and limited healthcare services. Incarcerated individuals are among vulnerable populations disproportionately affected by TB due to confined living conditions and delayed diagnosis. In Ethiopia, the prison setting provides an environment favorable to the rapid spread of TB and a threat to the outside community. Thus, this study aims to determine the prevalence of undiagnosed pulmonary tuberculosis (PTB) and its predictors among incarcerated individuals in Central Ethiopia.

**Methods:** A facility-based cross-sectional study was conducted from September to December 2023 among 363 selected incarcerated individuals in Central Ethiopia. Sociodemographic, clinical, and other risk-related data were collected using a structured questionnaire. Sputum samples were collected from incarcerated individuals with clinical symptoms of cough for two or more weeks and processed using GeneXpert MTB/RIF. The study was not formally powered to detect specific odds ratios for risk factor analysis; therefore, the associated predictors were explored through multivariable analysis and interpreted cautiously.

**Results:** In 3802 total incarcerated individuals in the region's prisons, 363 (9.5%) with clinical symptoms and 13 (0.34%) already on anti-TB treatment were identified. Among these 363 (9.5%) with clinical symptoms, 35 (9.64%) previously undiagnosed PTB cases were detected. Hence, the point prevalence of undiagnosed PTB among incarcerated individuals was 0.92% or 920 per 100,000 population (95% CI: 830–998/100,000), which is about 7.7 times higher than Ethiopia's general population (119/100,000). This previously undiagnosed PTB was associated with incarcerated individuals who had smoking, increased age, contact with coughing/TB patients, chronic illness comorbidity, overcrowding, and low BMI.

**Conclusion:** This study revealed a high point prevalence of undiagnosed PTB among incarcerated individuals. This mightily highlights that prisons are explicitly taken as a risky place for the transmission of PTB. Routine TB screening during prison entrance and periodical active case finding are highly recommended to identify missing people with TB who have a high spreading. After diagnosis, early treatment must be implemented to limit further transmission to incarcerated individuals and the surrounding community.

## 1. Introduction

Tuberculosis (TB) is a chronic airborne infectious disease primarily caused by *Mycobacterium tuberculosis* (MTB), typically affecting the lungs [1, 2]. Despite being preventable and curable, it remains a major public health problem globally, particularly in resource-limited settings where deprived ventilation, overcrowding, and limited healthcare services. According to the global TB report, it ranks as the second leading cause of death from infectious diseases, next to COVID-19 [3–5]. An estimated 10.6 million people yearly developed TB globally, with sub-Saharan Africa (SSA) including Ethiopia bearing a disproportionately high burden, particularly among adults in their most productive years [5, 6].

Incarcerated populations are particularly vulnerable to TB due to conditions like overcrowding, poor ventilation, limited access to health services, and a high prevalence of risk factors prior to incarceration such as HIV infection, malnutrition, and substance use that favor its transmission [7, 8]. Incarcerated people are likely to have TB prevalence rates many times higher than the general population, with undiagnosed cases posing a significant public health threat not only within the prison system but also to surrounding communities [8–10].

In Ethiopia, TB remains a major public health concern, ranking among the 30 high TB burden countries with a significant number of cases going undiagnosed and untreated each year [6, 11–13]. However, data on the burden of TB among incarcerated populations remain sparse and fragmented. Studies from different regions of the country have revealed alarmingly high rates of both active and latent [14–16]. TB among incarcerated individuals, with many cases remaining undiagnosed and untreated due to limited diagnostic capacity and weak prison health systems. Furthermore, the mobility of prison staff, visitors, and individuals released back into society underscores the potential for TB transmission beyond prison walls, representing an overlooked risk to community health [17–19].

Along with overcrowding situations, extended prison stays, low body mass index (BMI), previous TB treatment, increased age, alcohol intake, smoking, and HIV infection have been documented as the commonest identified risk factors for TB [14–16]. Because of this, prison is also unambiguously taken as a risky place for the spread of TB in low- and middle-income countries, where people are more likely than the general population to develop TB [17]. Prison staff, visitors, and family members may come into everyday contact with inmates [18]. TB can also continue to spread after a prisoner is freed if it is not properly diagnosed, treated, or connected to community-based care [19–21].

The rationale for focusing on undiagnosed PTB has been expanded to emphasize the lack of systematic screening, which contributes to diagnostic delays and continued transmission. Despite the availability of national guidelines recommending TB screening upon entry and periodic assessments within prison settings, these measures are inconsistently implemented due to systemic constraints [21, 22]. These findings in a substantial number of undiagnosed cases contribute to ongoing transmission within prisons and pose a threat to the wider community. Therefore, this study specifically focuses on identifying the burden and predictors of previously undiagnosed PTB.

## 2. Materials and Methods

### 2.1. Study Setting and Population

**Figure 1:**
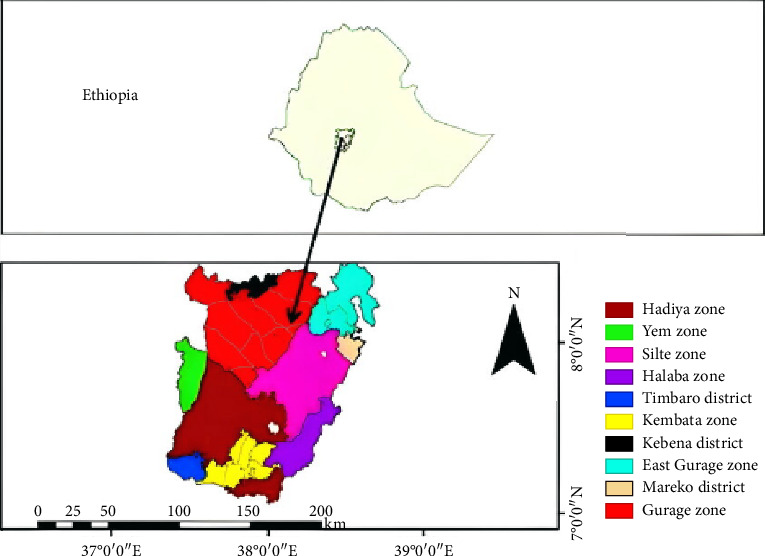
Map and position of study districts in Ethiopia.

The study was carried out at prisons found in the Central region of Ethiopia. The region is located in the Central part of the country and is a newly established region from the formerly Southern Nations Nationalities of People's Region (Figure 1). Administratively, the region has seven zones and two special woredas. Hossana town is a newly established administrative center of the region located 230 km from Addis Ababa, the capital city of the country. In the region, there are six zonal-level prisons with a total of 3802 incarcerated individuals at the time of data collection, but there are no data on the current prevalence of TB. There were a total of 975 incarcerated individuals in Hadiya, 480 in Halaba, 709 in Kembata, 510 in Gurage, 948 in Silte, and 180 in Yem Zonal prisons. All incarcerated individuals found in prisons of the region at the time of the study were the source population. Incarcerated individuals who had coughs for two or more weeks were included in the study.

### 2.2. Study Design and Period

A facility-based cross-sectional study was conducted from September to December 2023. All incarcerated individuals with a cough of 2 weeks and above were enrolled in the study.

### 2.3. Sample Size Determination

The sample size was calculated using a single population proportion formula that the calculation was primarily intended to estimate prevalence with acceptable relative precision with the assumptions for a 95% confidence level, 5% margin of error, and an expected prevalence of undiagnosed PTB of 12.1% based on prior studies among incarcerated populations. The study was not formally powered to detect specific odds ratios for risk factor analysis.

The classic formula for a single proportion in a cross-sectional study is as follows:

(1) $$\begin{array}{c} n=\frac{Z^{2}\cdot P\cdot \left(1-P\right)}{d2}, \end{array}$$

where

• *n* = required sample size
• *Z* = *Z*-score corresponding to the desired confidence level (1.96 for 95% confidence)
• *P* = expected prevalence of undiagnosed PTB, *P* = 0.121 (12.1%) based on literature or previous prison TB studies
• *d* = margin of error (precision), 0.05 (5%)

This gave an initial sample size of 165. After adjusting for a design effect of 2 and a 10% nonresponse rate, the final sample size was 363.

### 2.4. Sampling Procedures

To identify PTB possible cases, a mass screening approach was implemented in all prisons. This distributed an equal probability of picking eligible incarcerated individuals and minimized the chance of losing PTB possible cases. During this, every incarcerated individual was directly approached and screened by trained healthcare staff for the presence of a cough. This active case-finding method ensured the inclusion of all symptomatic individuals, rather than relying solely on self-reporting. For all incarcerated individuals who had coughs, a complete registration was done. All those who had a cough were interviewed and cross-examined to determine whether they fulfilled the inclusion criteria or not. All incarcerated individuals with complaints of cough for two or more weeks were included in the study. Among those complaints who were involuntarily to participate in the study, those who were critically ill, unable to provide information, and unable to produce sputum were excluded from the study. Finally, 363 study subjects who fulfilled the criteria and voluntarily participated were included in the study purposively.

### 2.5. Data Collection Tools and Process

A structured questionnaire was used to collect data on sociodemographic, clinical, and other risk factor variables. The questionnaire integrated the particular information of the participant's age, residence, gender, educational status, marital status, risk factors related to TB history or previous TB, length of incarceration, smoking, alcohol consumption, and other medical history, which were collected from the participants. The questions were asked in their languages. The physical characteristics of the study participants or BMI were also assessed. Underweight or low when the BMI was ≤ 18.5 kg/m^2^, and normal and above when the BMI was > 18.6 kg/m^2^ as recommended by the WHO [23].

### 2.6. Specimen Collection

About 3–5 mL of one early morning purulent sputum specimen was collected by skilled laboratory workers according to optional standards from each incarcerated individual with a complaint of cough for 2 weeks and above. The sputum specimen was collected in a sterile Falcon tube with a 30–50 mL volume since it was translucent and had walls that permitted labeling. The sputum was kept at a maximum temperature of 35°C for less than 3 days and in a 4°c refrigerator for 4–10 days. The sample was examined by Gene Xpert MTB/RIF by skilled laboratory personnel according to WHO guideline procedures. Sample reagent was added to the sputum sample at a 2:1 (V/V) ratio and mixed by handshaking [23]. The preparation was incubated at room temperature for 15 min. 2 mL of A well-kept sputum sample was moved to the cartridges using a sterile pipette, and the Gene Xpert machine was started. After 2 h, the results were interpreted and presented by the machine as MTB case identified/not identified.

### 2.7. Data Quality Assurance

All laboratory procedures were performed by following SOPs for GeneXpert MTB/RIF analysis. Sample processing control (SPC), internal quality control (IQC), and probe check control have been always done by the machine itself. External quality control is checked by recognized samples frequently for each GeneXpert facility for new batches. The quality controller for each LPA strip has five regulator zones, which are conjugate regulator, amplification regulator, and three locus regulator zones on rpoB, katG, and inhA promoter regions used to monitor the quality of the tests.

### 2.8. Screening for Chronic Illnesses

#### 2.8.1. Test for HIV

Among TB possible individuals, HIV pretest advising was given. Whole blood was collected from each incarcerated person. The serum was separated by centrifugation within 2 h of collection and stored at −20°C till used. HIV rapid testing was performed as per the recent national HIV-testing algorithm guideline procedures [24]. The test quality was checked by using previously known HIV-positive and HIV-negative samples. All equipment, procedures, and materials were adequately calibrated and maintained.

#### 2.8.2. Blood Glucose Measurement

The random blood glucose was detected by using a glucometer. For individuals with high random blood glucose detected, fasting blood glucose was measured by the hexokinase method within 2 h of venous blood extraction. Accordingly, diabetes was declared as fasting blood glucose ≥ 7.0 mmol/L or a history of Type 1 diabetes or Type 2 diabetes.

### 2.9. Data Entry and Analysis

Data were entered into an Epi-Data version 3.1 and exported to SPSS software Version 21 for analysis. To handle the variables, categorical variables were included as dummy variables and the continuous variables were checked for linearity with the log-odds. Missing data were also handled using complete case analysis. Sociodemographic and clinical data were analyzed and carried out by using percentages, frequencies, and mean with standard deviation (±SD) calculated to define the data. Univariable logistic regression was first analyzed individually for each potential independent variable to determine their crude association with the outcome variable (undiagnosed PTB). The variables with a *p* value < 0.25 in the univariable analysis were considered eligible for inclusion in the multivariable model. Multivariable logistic regression was used to identify factors influencing the occurrence of PTB and to assess their risk estimates. A stepwise backward elimination approach was used to identify the most parsimonious model. Variables were removed one at a time based on the highest *p* value. Multicollinearity was assessed after each removal for model's fit by using the variance inflation factor (VIF). The final model was taken from only those variables with statistical significance (*p* values < 0.05). The study was not formally powered to detect specific odds ratios for risk factor analysis, although exploratory logistic regression was conducted to identify associated factors; therefore, the observed associations in the multivariable analysis were interpreted with caution.

## 3. Results

### 3.1. Sociodemographic and Behavioral Characteristics of the Incarcerated Individuals

A total of 363 prison individuals who fulfilled the criteria (cough of 2 weeks and above) were included in the study, of whom 350 (96.4%) were males and 221 (60.9%) were married. The age of participants ranged from 18 to 76 years with a mean (±SD) age of 30 (±15.4) years having a normal distribution. The majority (57.7%) of the incarcerated individuals were from rural areas. At the time of data collection, about 83 (22.9%) and 139 (38.3%) of the study participants had a habit of cigarette smoking and khat chewing, respectively (Table 1).

### 3.2. Morbidity and Prison-Related Factors

Among study participants, for whom the BMI was measured, 213 (58.7%) had BMI ≥ 18.5 kg/m^2^ and 150 (41.3%) had a BMI lower than 18.5 kg/m^2^. Of all participants, 348 (95.9%) and 284 (78.2%) had no history of ever being diagnosed with TB and no history of hospitalization, respectively. About 315 (86.7%) prisoners had no history of cough symptoms before imprisonment, and the majority of prisoners, 326 (89.8%), had no history of contact with known TB patients. About 73 (20.1%) of the study subjects reported that 100 and above prisoners were living per room or in one room. 23 (6.3%) prisoners were HIV-positive (Table 2).

### 3.3. Prevalence of TB Among Incarcerated Individuals

Among 363 presumptive prison inmates with a cough duration of two weeks and above who were previously not diagnosed with TB, 35 (9.6%) were positive for MTB by GeneXpert MTB/RIF. This overall prevalence of undiagnosed PTB is significantly higher than the estimated national prevalence in the general population Ethiopia. This disparity underscores the heightened vulnerability and public health importance of TB control in prison settings.

Almost all of these presumptive inmates positive for MTB by GeneXpert MTB/RIF were males. Of these 35 PTB-identified cases, 17 (48.6%) were rural residents. The majority 24 (68.7%) of the PTB cases were married. The relative frequency of the PTB infection was higher (12.6%) in the age groups 35 years and above when compared with age groups less than 35 years 7.4% (Table 1). Of all undiagnosed PTB-diagnosed cases, 29 (82.9%) had TB symptoms after admission to the prison. Of these HIV-positive cases, 5 (21.7%) cases had undiagnosed TB co-infections. Among the prisoners, 40 (11.0%) were diagnosed as DM cases. Of these identified DM cases, 8 (20.0%) had undiagnosed TB co-infections (Table 2).

### 3.4. Factors Associated With Undiagnosed Active PTB

The study found that different variables were independently associated with undiagnosed PTB among the study participants. The prisoners aged 35 years and above had a 4.5 times higher risk of getting PTB [AOR = 4.53; 95% CI (2.52, 11.31)] than the younger ages (less than 35 years). The risk of getting undiagnosed active PTB was greater than 4.2 times more likely among incarcerated individuals who had the habit of cigarette smoking before incarceration [AOR = 4.24; 95% CI (2.81–9.99)] compared to nonsmokers. History of khat chewing [AOR = 3.22; 95% CI (1.06, 9.74)] and contact history with coughing or TB patients [AOR = 3.32; 95% CI (1.29, 8.59)] were also independently associated with increased occurrence of active undiagnosed PTB.

Among prisoners with low BMI (< 18.5 kg/m^2^), the risk of getting active undiagnosed PTB was about 5 times higher compared to increased BMI [AOR = 5.02; 95% CI (1.16, 7.31)]; likewise, those who have a longer duration of stay in present prison were nearly 3.6 times more likely develop PTB than those who have a shorter duration of stay in present prison [AOR = 3.65; 95% CI (1.22, 10.90)] (Table 3).

## 4. Discussion

TB remains a key public health risk worldwide, predominantly in the SSA region [3]. In studies done in these regions [14], an estimated pooled prevalence of PTB among prisoners in SSA is significantly higher than in the general population and is many times higher than the national prevalence [15, 25]. This aligns with our findings and underscores the disproportionately high TB burden in prison populations. In the present study, consistently the point prevalence of undiagnosed PTB among incarcerated individuals in prisons of the central region of Ethiopia was very high, 0.92% (35/3802) or 920 cases per 100,000 [95% CI (830–992)] which was many times higher than estimated point prevalence in Ethiopia, which was nearly 119 per 100,000 [95% CI (91–241)]; however, it varies by regions and it was also about sixfold higher (nearly 148 per 100,000) than in general population in Southern Ethiopia [26]. This increased incidence of TB in incarcerated individuals could be likely the fact that they are an ignored population in the case of early screening, diagnosis, and treatment of TB in a high-risk population.

The observed point prevalence of undiagnosed PTB among incarcerated individuals with a cough of duration more than 2 weeks was also high, 9.6% [95% CI (5.1%, 12.2%)], which is significantly higher than in the general population of the study area, 4.6% [26]. The possible reason for this might be due to differences in study populations and study settings. In addition, this could also be the use of GeneXpert contributed to improved case findings that GeneXpert is more accurate and reliable than sputum smear microscopy in foreseeing PTB [27]. Furthermore, new cases of PTB detected during the time of data collection could indicate that undiagnosed PTB in the prisons in the study area is the source of infection for other inmates. Therefore, in this study area, there is a need for regular screening of TB during entrance and departure on a routine basis.

On multivariate analysis, incarcerated individuals who had a history of “khat” chewing before incarceration had about 3 times increased risk of developing PTB [AOR = 3.22; 95% CI (1.06, 9.74)] than those who had not chewed “khat” which is consistent with studies reported in other previous study done in Ethiopia [28, 29]. This similarity could be due to most khat chewers practicing in confined areas for extended periods, which increases the contact time if there is an infected incarcerated individual among them. Sharing prison cells or being incarcerated with known TB in the same room was also significantly associated with PTB infection [AOR = 3.32; 95% CI (1.29, 8.59)] which is in line with the study done in Eastern [28] and Western Ethiopia [30]. This is because when the noninfected incarcerated individuals share a room, it is easy to receive and inhale the droplets from infected inmates.

In this study, nutritional status with low BMI was significantly related to PTB incidence [AOR = 5.02; 95% CI (1.16, 7.31)]. BMI < 18.5 kg/m^2^ had a pertinent effect on PTB as compared to those incarcerated individuals with BMI ≥ 18.5 kg/m^2^, which is consistent with studies done in Ethiopia [31–34] and other countries (39–42) that reported low BMI (i.e. ≤ 18.5 kg/m^2^) as the risk factor for PTB. The possible reason for this could be that malnutrition is similarly documented as a risk factor for the re-activation of latent TB, compromising host immunity, increasing susceptibility to TB infection, and its advancement to disease. Moreover, the bidirectional relationship between low BMI and TB, conceding malnutrition as both a risk factor for TB infection and a consequence of active disease. This suggestion is supported by previous studies in Northern Ethiopia [35] and in Southern Ethiopia, Wolaita Sodo [36], and this relation is also bidirectional as TB can cause or predispose to malnutrition. Thus, when comparing persons with and without active PTB in this cross-sectional study regarding their nutritional status, a causal effect cannot be allocated to malnutrition, whereas it emanated as an important predictor in the multivariate analysis.

The present study revealed that most of the undiagnosed PTB-detected incarcerated individuals developed PTB symptoms after they met the incarcerated individuals. Although there was no observed significant association between PTB positivity and its symptoms in this study, there are multiple studies that presented significant associations [29, 34, 35]. The SSA prison studies reported a variety of symptoms like weight loss and loss of appetite that had a significant independent contribution to a diagnosis of smear-positive PTB [14, 37]. Similarly, this in combination with long cough duration shows a prolonged interval of time before patients get diagnosed and treated rendering the smear-positive prisoners to spread the infection to many others [38]. This could be increased by the environment of the cells shared by the incarcerated individuals. The cells in the study area were poorly ventilated having no windows or single windows many of them even not open during the day and housed more than one hundred incarcerated individuals (with an average of 150 incarcerated individuals per cell) who contacted the whole day with incarcerated individuals from other cells in delimited spaces. The extended stay of the incarcerated individuals in the prisons might have rendered the prisons to serve as a reservoir pool of TB transmission. The period left to stay in the prisons for most of the incarcerated individuals with PTB was greater than a year which could advance augment the spread of TB.

Importantly, several variables in this study were not statistically significant but are still worth discussing due to their relevance in the TB transmission context. Although no significant association was found between educational level and undiagnosed PTB in this study, low literacy levels have been previously linked with reduced awareness of TB symptoms and transmission, contributing to delays in diagnosis and care [39]. Educational interventions within prison settings remain crucial for early case identification and improving treatment outcomes. Similarly, the duration of cough among symptomatic inmates was not significantly associated with PTB positivity. However, a persistent cough remains a key symptom of concern. The lack of statistical significance could be related to the nonspecific nature of the cough or recall bias in reporting duration. Nonetheless, any inmate reporting a cough of more than 2 weeks should still be promptly investigated for TB as per WHO guidelines [3].

While duration of incarceration was not a significant predictor in this study, longer prison stays are commonly associated with increased TB exposure and risk of infection, particularly in overcrowded and poorly ventilated environments [40]. The lack of significance in our analysis could be due to variability in prison conditions or early-stage infections not yet manifesting detectable symptoms. Hospitalization history was not significantly associated with undiagnosed PTB, but given that healthcare facilities can also be settings of TB transmission, especially when infection prevention measures are weak, this variable remains important in TB epidemiology [41].

Although gender and marital status were also not a statistically significant predictors in our analysis, the predominance of male inmates might obscure potential gender-related vulnerabilities. Some studies have indicated that women may be more susceptible to delayed TB diagnosis due to stigma and access barriers, even though their overall prevalence in prison populations is lower [42]. The study suggested that the marital status may influence social support structures, which in turn can affect health-seeking behavior and adherence to treatment [43]. Incarcerated individuals who are single or divorced might experience isolation, exacerbating mental health challenges that deter them from seeking timely medical care.

### 4.1. Limitations

This study has certain limitations that should be considered when interpreting the findings. First, the study only included incarcerated individuals who exhibited a cough lasting 2 weeks or more, potentially underestimating the true prevalence of TB by excluding asymptomatic individuals. Second, the use of GeneXpert for TB diagnosis, while highly sensitive and specific, may still miss some cases, especially among individuals with low bacillary load. Third, the study was limited to selected prisons in Central Ethiopia, which may affect the generalizability of the findings to other regions of the broader incarcerated population or other high-risk groups. Fourth, it is not possible to determine a cause-and-effect relationship between risk factors and TB status because the exposure and outcome were assessed simultaneously. In addition, the findings reflect survival rather than true incidence, potentially underrepresenting individuals with rapidly progressing or self-resolving. Additionally, due to resource constraints, culture confirmation was not performed, which might have further improved diagnostic accuracy. Lastly, the potential for underreporting or misclassification of individuals already on TB treatment could influence the estimation of the true prevalence of undiagnosed TB cases. Despite these limitations, the study provides important evidence on the overlooked prevalence of TB in a high-risk population and underscores the urgent need for enhanced TB screening and control strategies in correctional facilities.

## 5. Conclusion and Recommendation

In this study, we observed that the burden of PTB among incarcerated individuals was very high when compared with the general population. There was also a high burden of active undiagnosed PTB cases in the prisons. This indicates prisons are specifically taken as risky places for the transmission of TB for incarcerated individuals, prison staff, visitors, and a large number of family members who come into everyday contact with incarcerated individuals. They also continue to spread TB through various means after the incarcerated individuals are freed if it is not properly diagnosed, treated, or connected to community-based care. History of cigarette smoking before imprisonment, history of contact with known TB patients, presence of chronic diseases, and lower BMI were identified as risky for active transmission of undiagnosed PTB and put the prison inhabitants at increased risk of developing PTB. Therefore, the findings of this study underscore the urgent need to strengthen TB control measures in prison settings in Ethiopia and similar contexts.

Based on these findings, more TB patients remain missed and unreached, impacting negatively on health outcomes. We would like to recommend that the concerned bodies' TB case-finding approaches should be revised and advanced approaches and tools to identify missing people with TB should be scaled up. The screening of incarcerated individuals during entry, periodic screening for symptomatic, and exit screening to identify early undiagnosed infectious cases, prevents further delay in diagnosis and reduces the prolonged spread of TB in the prisons and surrounding community. Finally, priority should be given to prisons for active case finding, training for incarcerated individuals and prison staff on TB infection prevention, and continuous health information on the mode of PTB control and its prevention mechanisms. Additionally, we would like to recommend the concerned bodies' on the apparent gaps between policy and practice that highlighting the prison authorities should fully implement the recommended Ethiopia's national routine TB screening protocols that integrate the TB programs with general prison health services and linkage to community-based TB program upon postrelease are critical to preventing ongoing community transmission beyond prison walls. Finally, we would like to recommend that operational research should be done to assess the feasibility and cost-effectiveness of innovative screening tools, such as digital chest X-rays with computer-aided detection, which could enhance early TB case detection in resource-limited prison environments.

## Figures and Tables

**Table 1:** Sociodemographic and behavioral characteristics of incarcerated individuals in the central region of Ethiopia, 2023 (*N* = 363).

Variable	Label	Number (%)	PTB status	
			Has PTB	Has no PTB
Age	18–24	94 (29.2)	6 (6.4)	88 (93.6)
	25–34	110 (31.1)	9 (8.2)	101 (91.8)
	35–44	86 (15.1)	11 (12.8)	75 (87.2)
	≥ 45	73 (25.1)	9 (12.3)	64 (87.7)

Gender	Female	13 (3.6)	1 (7.7)	12 (92.3)
	Male	350 (96.4)	34 (9.7)	316 (90.3)

Marital status	Single	130 (35.8)	10 (7.7)	120 (92.3)
	Married	221 (60.9)	24 (10.9)	197 (89.1)
	Divorced	12 (3.3)	1 (8.3)	11 (91.7)

Level of education	Illiterate	82 (22.6)	5 (6.1)	77 (93.9)
	Primary and secondary	159 (43.8)	17 (10.7)	142 (89.3)
	College and above	122 (33.6)	2 (1.6)	120 (98.4)

Residence	Rural	213 (57.7)	17 (8.0)	196 (92.0)
	Urban	150 (42.3)	18 (12.0)	132 (88.0)

Cigarette smoking	No	280 (77.1)	21 (7.5)	259 (92.5)
	Yes	83 (22.9)	14 (16.9)	69 (83.1)

Khat chewing	No	224 (61.7)	16 (7.2)	208 (92.8)
	Yes	139 (38.3)	19 (13.7)	120 (86.3)

**Table 2:** Morbidity and prison-related factors of prisoners in the central region of Ethiopia, 2023.

Variable	Label	Number (%)	PTB status	
			Has PTB	Has no PTB
Number of incarcerate individuals per room or cell	< 100	290 (79.9)	25 (8.2)	265 (91.4)
	≥ 100	73 (20.1)	10 (13.7)	63 (86.3)

Duration of cough	< 4 weeks	150 (41.3)	6 (4.0)	144 (96.0)
	≥ 4 weeks	213 (58.7)	29 (13.6)	184 (86.4)

Length of staying in current prison	≤ 24 months	256 (70.5)	22 (8.6)	234 (91.4)
	> 24 months	107 (29.5)	13 (12.1)	94 (87.9)

Incarceration in another prison	No	344 (94.8)	32 (9.3)	312 (90.7)
	Yes	19 (5.2)	3 (15.8)	16 (84.2)

Cough symptoms before incarceration	Yes	48 (13.2)	6 (12.5)	42 (87.5)
	No	315 (86.7)	29 (9.2)	286 (90.8)

Had ever diagnosed for TB	No	348 (95.9)	33 (9.5)	315 (90.1)
	Yes	15 (4.1)	2 (13.3)	13 (86.7)

Hospitalization	No	284 (78.2)	25 (8.8)	259 (91.2)
	Yes	79 (21.8)	10 (12.7)	69 (87.3)

Contact with coughing/known TB patient	No	326 (89.8)	27 (8.3)	299 (91.2)
	Yes	37 (10.2)	8 (21.6)	29 (78.4)

HIV test result	Positive	23 (6.3)	5 (21.7)	18 (78.3)
	Negative	302 (83.2)	24 (7.9)	278 (92.1)
	Not tested	38 (10.5)	6 (15.8)	32 (84.2)

DM	No	323 (89.0)	27 (8.4)	296 (91.4)
	Yes	40 (11.0)	8 (20.0)	32 (80.0)

Body mass index	< 18.5	150 (41.3)	19 (12.7)	131 (87.3)
	≥ 18.5	213 (58.7)	16 (7.5)	197 (92.5)

*Note:* (*N* = 363).

**Table 3:** Logistic regression analysis of risk factors associated with undiagnosed PTB among prisoners of entral region of Ethiopia, 2023.

Characteristics	PTB status		COR (95% CI)	AOR (95% CI)
	Positive	Negative		
Age				
18–34	15	189	1	1
≥ 35	20	139	1.82 (1.32–8.71)^∗^	4.5 (2.52–11.31)^∗∗^
Gender				
Male	34	316	1.29 (0.83–5.11)	1.35 (0.94–4.23)
Female	1	12	1	1
Marital status				
Single	10	120	1	1
Married	24	197	1.46 (1.26–6.24)^∗^	2.01 (0.82–6.04)
Divorced	1	11	1.01 (0.42–4.75)	1.51 (0.73–3.40)
Level of education				
Illiterate	5	77	1.84 (1.23–4.01)^∗^	1.02 (0.96–5.06)
Primary and secondary	17	142	0.26 (0.18–3.12)	0.73 (0.48–3.86)
College and above	2	120	1	1
Residence				
Rural	17	196	1.57 (1.38–7.86)^∗^	2.07 (0.81–6.62)
Urban	18	132	1	1
Cigarette smoking				
No	21	259	1	1
Yes	14	69	2.50 (1.01–9.15)^∗^	4.24 (2.81–9.99)^∗∗^
Khat chewing				
No	16	208	1	1
Yes	19	120	2.06 (1.51–8.71)^∗^	3.22 (1.06, 9.74)^∗∗^
Length of staying				
< 24 weeks	22	234	1	1
≥ 24 weeks	13	94	1.47 (1.22–10.90)^∗^	3.65 (2.22–11.13)
Cough symptom before incarceration				
No	6	42	1	1
Yes	29	286	1.41 (1.01–2.71)^∗^	1.87 (1.44–6.33)
Duration of cough				
< 4 weeks	6	144	1	1
≥ 4 weeks	29	184	3.78 (2.14–9.23)^∗^	3.05 (0.87–8.82)
Hospitalization:				
No	25	259	1	1
Yes	10	69	1.50	1.01 (0.55–3.21)
HIV test result				
Positive	5	18	3.22 (3.01–9.59)^∗^	8.32 (1.29–13.59)^∗∗^
Negative	24	278	1	1
Diagnosed DM				
No	27	296	1	1
Yes	8	32	2.74 (2.07–6.71)^∗^	4.52 (3.21–12.38)^∗∗^
Contact with coughing/known TB patient				
No	27	299	1	1
Yes	8	29	3.05 (1.57–9.05)^∗^	3.9 (1.44–9.63)^∗∗^
Number of prisoners per cell:				
≤ 100	25	265	1	1
> 100	10	63	1.68 (1.09–11.07)^∗^	5.02 (3.11–10.29)^∗∗^
Body mass index				
< 18.5	19	131	1.79 (1.02–6.02)^∗^	5.02 (1.16, 7.31)^∗∗^
≥ 18.5	16	197	1	1

*Note:* For multivariate analysis, the candidate variable is at *p* < 0.25. 1, reference.

Abbreviations: AOR, adjusted odds ratio; CI, confidence interval; COR, crude odds ratio.

^∗^Significant variable significant.

^∗∗^Highly significant variable by the multivariate analysis at *p* < 0.05.

## Data Availability

The data that support the findings of this study are available from the corresponding author upon reasonable request.

## References

[1] Yadav K., Prakash S. (2017). Tuberculosis: An Airborne Disease. *Global Journal of Medical Research*.

[2] Talip B. A., Sleator R. D., Lowery C. J., Dooley J. S., Snelling W. J. (2013). An Update on Global Tuberculosis (TB). *Infectious Diseases: Research and Treatment*.

[3] World Health Organization (2023). *Global Tuberculosis Report 2023*.

[4] Jeong Y. J., Lee K. S. (2008). Pulmonary Tuberculosis: Up-To-Date Imaging and Management. *American Journal of Roentgenology*.

[5] Dye C., Scheele S., >Dolin P., Pathania V., Raviglione M. C. (1999). Global Burden of Tuberculosis: Estimated Incidence, Prevalence, and Mortality by Country. *JAMA*.

[6] Cords O., Martinez L., Warren J. L. (2021). Incidence and Prevalence of Tuberculosis in Incarcerated Populations: A Systematic Review and Meta-Analysis. *The Lancet Public Health*.

[7] Bhargava A., Bhargava M., Juneja A. (2021). Social Determinants of Tuberculosis: Context, Framework, and the Way Forward to Ending TB in India. *Expert Review of Respiratory Medicine*.

[8] Naning H. *Modeling the Impact of Tuberculosis Control Measures in a Highly Endemic and an Overcrowded Prison*.

[9] Sequera G., Estigarribia G., Walter K. S., Lopez R., Andrews J., Croda J. (2023). Tuberculosis in Prisons: A Growing Global Health Concern. *The Challenge of Tuberculosis in the 21st Century: ERS Monograph*.

[10] Naik D. K., Fly V., Mall A., Palace B. H. (2019). The Problems of Prisoners: An Analysis. *International Journal of Research and Analytical Reviews*.

[11] Sadiq S., Parveen R. (2014). Imprisonment and Health: Issues and Concerns. *International Journal of Advanced Research in Management and Social Sciences*.

[12] Nguipdop-Djomo P., Rodrigues L. C., Abubakar I., Mangtani P. (2020). Small-Area Level Socio-Economic Deprivation and Tuberculosis Rates in England: An Ecological Analysis of Tuberculosis Notifications Between 2008 and 2012. *PLoS One*.

[13] Behera D., Pannu V. P., Behera R. K. (2022). National TB Elimination Programme–Can it End TB in India by 2025: An Appraisal. *Indian Journal of Chest Diseases and Allied Sciences*.

[14] Sisay Asgedom Y., Ambaw Kassie G., Melaku Kebede T. (2023). Prevalence of Tuberculosis Among Prisoners in Sub-Saharan Africa: A Systematic Review and Meta-Analysis. *Frontiers in Public Health*.

[15] Melese A., Demelash H. (2017). The Prevalence of Tuberculosis Among Prisoners in Ethiopia: A Systematic Review and Meta-Analysis of Published Studies. *Archives of Public Health*.

[16] Rao V. G., Bhat J., Yadav R., Sharma R. K., Muniyandi M. (2018). A Comparative Study of the Socio-Economic Risk Factors for Pulmonary Tuberculosis in the Saharia Tribe of Madhya Pradesh, India. *Transactions of the Royal Society of Tropical Medicine & Hygiene*.

[17] Biadglegne F., Rodloff A. C., Sack U. (2015). Review of the Prevalence and Drug Resistance of Tuberculosis in Prisons: A Hidden Epidemic. *Epidemiology and Infection*.

[18] Pescarini J. M., Rodrigues L. C., Gomes M. G., Waldman E. A. (2017). Migration to Middle-Income Countries and Tuberculosis-Global Policies for Global Economies. *Globalization and Health*.

[19] Beaudry G., Zhong S., Whiting D., Javid B., Frater J., Fazel S. (2020). Managing Outbreaks of Highly Contagious Diseases in Prisons: A Systematic Review. *BMJ Global Health*.

[20] Glaser J. B., Greifinger R. B. (1993). Correctional Health Care: A Public Health Opportunity. *Annals of Internal Medicine*.

[21] Bagcchi S. (2023). Who's Global Tuberculosis Report 2022. *The Lancet Microbe*.

[22] Chunrong L. U., Hongxia F. A., Puxuan L. U., Lecai J. (2021). The Global Tuberculosis Report 2021: Key Data Analysis for China and the Global World. *Electronic Journal of Emerging Infectious Diseases*.

[23] Addis Z., Adem E., Alemu A. (2015). Prevalence of Smear Positive Pulmonary Tuberculosis in Gondar Prisoners, North West Ethiopia. *Asian Pacific Journal of Tropical Medicine*.

[24] World Health Organization (2014). *Xpert MTB/RIF Implementation Manual: Technical and Operational ‘How-To’; Practical Considerations*.

[25] Lukoye D., Ssengooba W., Musisi K. (2015). Variation and Risk Factors of Drug Resistant Tuberculosis in Sub-Saharan Africa: A Systematic Review and Meta-Analysis. *BMC Public Health*.

[26] Datiko D. G., Guracha E. A., Michael E. (2019). Sub-National Prevalence Survey of Tuberculosis in Rural Communities of Ethiopia. *BMC Public Health*.

[27] Shah S. W., Ullah R., Raja S. K. (2019). Diagnostic Accuracy of Genexpert and Sputum Zeil Nelson Staining in Predicting Tuberculosis Taking Sputum Culture as Gold Standard. *Pakistan Journal of Chest Medicine*.

[28] Ephrem K., Dessalegn Z., Etana B., Shimelis C. (2022). Prevalence of Pulmonary Tuberculosis and Associated Factors Among Prisoners in Western Oromia, Ethiopia: A Cross-Sectional Study. *Journal of Science, Technology and Arts Research*.

[29] Duressa T. A., Mersha M. A., Assefa D., Klinkenberg E. Prevalence and Associated Risk Factors of Pulmonary Tuberculosis Among Prisoners in Benishangul Gumuz Region, Ethiopia.

[30] Dibissa K. E., Waktole Z. D., Tolessa B. E. (2019). Prevalence of Pulmonary Tuberculosis and Associated Factors Among Prisoners in Western Oromia, Ethiopia: A Cross-Sectional Study. *bioRxiv*.

[31] Hussien B., Hussen M. M., Seid A., Hussen A. (2019). Nutritional Deficiency and Associated Factors Among New Pulmonary Tuberculosis Patients of Bale Zone Hospitals, Southeast Ethiopia. *BMC Research Notes*.

[32] Seid G., Ayele M. (2020). Undernutrition and Mortality Among Adult Tuberculosis Patients in Addis Ababa, Ethiopia. *Advances in preventive medicine*.

[33] Endalkachew K., Ferede Y. M., Derso T., Kebede A. (2022). Prevalence and Associated Factors of Undernutrition Among Adult TB Patients Attending Amhara National Regional State Hospitals, Northwest Ethiopia. *Journal of Clinical Tuberculosis and Other Mycobacterial Diseases*.

[34] Wondmieneh A., Gedefaw G., Getie A., Demis A. (2021). Prevalence of Undernutrition Among Adult Tuberculosis Patients in Ethiopia: A Systematic Review and Meta-Analysis. *Journal of Clinical Tuberculosis and Other Mycobacterial Diseases*.

[35] Brhane T., Merga H., Ayele L., Gemeda D. H. (2021). Undernutrition Among Tuberculosis Patients on Directly Observed Short-Course Therapy: An Epidemiological Study from Northern Ethiopia. *Nutrition and Dietary Supplements*.

[36] Duko B., Bedaso A., Ayano G., Yohannis Z. (2019). Perceived Stigma and Associated Factors Among Patient With Tuberculosis, Wolaita Sodo, Ethiopia: Cross-Sectional Study. *Tuberculosis research and treatment*.

[37] Bernard C., Dabis F., de Rekeneire N. (2017). Prevalence and Factors Associated With Depression in People Living With HIV in Sub-Saharan Africa: A Systematic Review and Meta-Analysis. *PLoS One*.

[38] Brown E. (2020). A Systematic Review of the Effects of Prison Segregation. *Aggression and Violent Behavior*.

[39] Legesse M., Ameni G., Mamo G. (2010). Knowledge and Perception of Pulmonary Tuberculosis in Pastoral Communities in the Middle and Lower Awash Valley of Afar Region, Ethiopia. *BMC Public Health*.

[40] Aerts A., Hauer B., Wanlin M., Veen J. (2006). Tuberculosis and Tuberculosis Control in European Prisons. *International Journal of Tuberculosis & Lung Disease*.

[41] Ulo A. O. (2020). Nosocomial Tuberculosis Transmission in African Healthcare Settings: A Systematic Review. *PLoS One*.

[42] Zellweger J. P., Coulon P. (2015). Tuberculosis and Prisons. *American Journal of Respiratory and Critical Care Medicine*.

[43] Sreeramareddy C. T. (2014). Stigma and Delay in Seeking Care Among Tuberculosis Patients in India. *Journal of Biosocial Science*.

[44] Mogoere S. P. (2013). *Risk Factors Associated With Tuberculosis at Mangaung Correctional Centre: Retrospective Analysis*.

[45] Van Hout M. C., Mhlanga-Gunda R. (2019). Prison Health Situation and Health Rights of Young People Incarcerated in Sub-Saharan African Prisons and Detention Centres: A Scoping Review of Extant Literature. *BMC International Health and Human Rights*.

[46] Shanks L., Siddiqui M. R., Kliescikova J. (2015). Evaluation of HIV Testing Algorithms in Ethiopia: The Role of the Tie-Breaker Algorithm and Weakly Reacting Test Lines in Contributing to a High Rate of False Positive HIV Diagnoses. *BMC Infectious Diseases*.

[47] Cho S. H., Lee H., Kwon H. (2022). Association of Underweight Status With the Risk of Tuberculosis: A Nationwide Population-Based Cohort Study. *Scientific Reports*.

[48] Musuenge B. B., Poda G. G., Chen P. C. (2020). Nutritional Status of Patients With Tuberculosis and Associated Factors in the Health Centre Region of Burkina Faso. *Nutrients*.

[49] Nthiga I., Mbithe D., Mugendi B. J., Wambui T. *The Nutritional Status of Pulmonary Tuberculosis Patients Aged 25-44 Years Attending Tuberculosis Clinic at Lodwar County and Referral Hospital*.

[50] Mburu J. W., Kingwara L., Ester M., Andrew N. (2018). Prognostic Factors Among TB and TB/DM Comorbidity Among Patients on Short Course Regimen Within Nairobi and Kiambu Counties in Kenya. *Journal of Clinical Tuberculosis and Other Mycobacterial Diseases*.

[51] Elfaky I. O., Merghani T. H., Elmubarak I. A., Ahmed A. H. (2023). Nutritional Status and Patterns of Anemia in Sudanese Adult Patients With Active Pulmonary Tuberculosis: A Cross-Sectional Study. *The International Journal of Mycobacteriology*.

